# The Contribution of Normal Pregnancy to Eclampsia

**DOI:** 10.1371/journal.pone.0133953

**Published:** 2015-07-28

**Authors:** Abbie Chapman Johnson, Keith J. Nagle, Sarah M. Tremble, Marilyn J. Cipolla

**Affiliations:** 1 Department of Neurological Sciences, University of Vermont College of Medicine, Burlington, Vermont, 05405, United States of America; 2 Department of Obstetrics, Gynecology, and Reproductive Sciences, University of Vermont College of Medicine, Burlington, Vermont, 05405, United States of America; 3 Department of Pharmacology, University of Vermont College of Medicine, Burlington, Vermont, 05405, United States of America; University of Rome Tor Vergata, ITALY

## Abstract

Eclampsia, clinically defined as unexplained seizure in a woman with preeclampsia, is a life threatening complication unique to the pregnant state. However, a subpopulation of women with seemingly uncomplicated pregnancies experience de novo seizure without preeclamptic signs or symptoms, suggesting pregnancy alone may predispose the brain to seizure. Here, we hypothesized that normal pregnancy lowers seizure threshold and investigated mechanisms by which pregnancy may affect seizure susceptibility, including neuroinflammation and plasticity of gamma-aminobutyric acid type A receptor (GABA_A_R) subunit expression. Seizure threshold was determined by quantifying the amount of pentylenetetrazole (PTZ) required to elicit electrical seizure in Sprague Dawley rats that were either nonpregnant (Nonpreg, n = 7) or pregnant (Preg; d20, n = 6). Seizure-induced vasogenic edema was also measured. Further, activation of microglia, a measure of neuroinflammation (n = 6-8/group), and GABA_A_R δ- and γ2-subunit protein expression in the cerebral cortex and hippocampus (n = 6/group) was determined. Seizure threshold was lower in Preg compared to Nonpreg rats (36.7±9.6 vs. 65.0±14.5 mg/kg PTZ; p<0.01) that was associated with greater vasogenic edema formation (78.55±0.11 vs. 78.04±0.19% water; p<0.05). The % of active microglia was similar between groups; however, pregnancy was associated with downregulation of cortical GABA_A_R-δ and hippocampal GABA_A_R-γ2 expression. Overall, pregnancy appears to be a state of increased seizure susceptibility that is not due to neuroinflammation, but rather is associated with reduced expression of GABA_A_R subunits and greater edema. Understanding neurophysiological changes occurring in normal pregnancy could allow for better prevention and management of de novo seizure, including pathologic states such as eclampsia.

## Introduction

Preeclampsia, defined as the new onset of hypertension and proteinuria after the 20^th^ week of gestation, is a life-threatening complication of pregnancy that afflicts 1–7% of all pregnancies [1–4]. Numerous organs are affected by preeclampsia, including the brain in the form of eclampsia [5–9]. Eclampsia is the appearance of unexplained seizure in a woman with preeclampsia and is one of the most dangerous complications of pregnancy [4]. Eclampsia is a leading cause of maternal and fetal morbidity and mortality worldwide that accounts for greater than 50,000 maternal deaths each year [6, 7, 10]. While eclampsia by definition is restricted to women with preeclampsia, seizure during pregnancy does not appear to be a progression from severe preeclampsia to eclampsia [11–13]. In fact, de novo seizure has been reported to occur in 38% of seemingly uncomplicated pregnancies, without hypertension and proteinuria, or the diagnosis of preeclampsia [12]. The finding that de novo seizure occurs in the absence of preeclampsia suggests that pregnancy alone may be a state of increased seizure susceptibility. In addition, women who develop eclampsia are by definition normotensive and asymptomatic prior to pregnancy, with no known underlying conditions contributing to seizure onset, supporting the concept that pregnancy alone may predispose the brain to seizure independently of preeclampsia.

It is well-established that fluctuations in neurosteroids occur during the menstrual cycle, and to a greater extent during pregnancy, that can affect neuronal excitability [14–16]. Specifically, increases in progesterone metabolites, including allopregnanolone, act as positive allosteric modulators of gamma-aminobutyric acid type A receptors (GABA_A_Rs), the main inhibitory neurotransmitter receptors in the brain, and decrease neuronal excitability [16]. GABA_A_Rs consist of several subunits, however, the main binding site and therefore site of action of allopregnanolone is on the δ-subunit [17]. δ-subunit-containing GABA_A_Rs (GABA_A_R-δ) are located extrasynaptically and are involved in tonic inhibition throughout the brain [16]. Neuroactive steroids such as allopregnanolone reduce neuronal excitability selectively through actions at GABA_A_R-δ; however, GABA_A_R-δ are downregulated in response to increased allopregnanolone to maintain the excitatory/inhibitory balance [16, 17]. Plasticity of the δ-subunit of GABA_A_Rs occurs in the brain during pregnancy, and is an adaptation that likely functions to maintain a steady state of excitability in the face of increased neurosteroids [17, 18]. In fact, brain slices from pregnant mice were found to be hyperexcitable due to the downregulation of the δ-subunit that was normalized by the presence of allopregnanolone [18]. While these in-vitro studies suggest that the brain is hyperexcitable during pregnancy, it is less clear if the brain is more susceptible to seizure under normal physiological conditions of pregnancy where naturally circulating neurosteroids are present. Further, synaptic γ2-subunit-containing GABA_A_Rs (GABA_A_R-γ2) are involved in phasic inhibition and have also been shown to decrease expression in the brain during pregnancy [19]. Although synaptic GABA_A_R-γ2 are not affected by neurosteroids [16], pregnancy-induced changes in their expression may also contribute to increased seizure susceptibility during pregnancy.

One mechanism by which brain excitability may be affected during pregnancy is through peripheral inflammation. Pregnancy is considered a state of mild peripheral inflammation and peripheral inflammation has been shown to cause neuroinflammation through the activation of microglia, the resident immune cells in the brain [20, 21]. When microglia become active, they aid in clearance of debris and play a reparative role in the brain; however, for unknown reasons they can become cytotoxic [22]. Active microglia secrete proinflammatory cytokines and increase local neuronal excitability by simultaneously promoting the endocytosis of GABA_A_Rs and exocytosis of excitatory α-amino-3-hydroxy-5-methyl-4-isoxazolepropionic acid (AMPA) receptors [21, 23]. In fact, neuroinflammation has recently been shown to be present in a rat model of preeclampsia that was associated with decreased seizure threshold [24]. Whether neuroinflammation is present during normal pregnancy is unknown, however, it may be one mechanism by which the pregnant state is more susceptible to seizure. We hypothesized that pregnancy is a state of increased seizure susceptibility due to decreased expression of GABA_A_Rs and/or neuroinflammation that acts to lower seizure threshold.

Onset of eclampsia may also be related to loss of cerebral blood flow autoregulation and decreased cerebrovascular resistance that increases pressure on the microcirculation and subsequent vasogenic edema formation [25]. In fact, vasogenic edema is present in ~ 90% of women with eclampsia, as assessed by diffusion weighted MRI [26, 27]. Further, vasogenic edema formation occurs to a greater extent during pregnancy under pathologic conditions such as acute hypertension, suggesting pregnancy predisposes the brain to edema [28, 29]. Thus, we hypothesized that the maternal brain is more susceptible to seizure-induced vasogenic edema formation than in the nonpregnant state. Understanding if the maternal brain is more susceptible to seizure-induced edema may lead to a greater understanding of the pathophysiological process of de novo seizure during pregnancy.

## Methods

### Animals and ethics statement

All experiments were conducted using virgin, nonpregnant (Nonpreg) or timed-pregnant (Preg) Sprague Dawley rats that were 14–16 weeks old (Charles River, Canada). Pregnant rats were used experimentally late in gestation (day 20 of a 22 day gestation), a time point when eclampsia occurs most often [12]. Preg rats do not exhibit preeclamptic-like symptoms, as it has previously been reported that Nonpreg and Preg rats have similar blood pressures and urinary protein excretion [30, 31]. All rats were housed singly in the University of Vermont Animal Care Facility, an Association for Assessment and Accreditation of Laboratory Animal Care International accredited facility. All procedures were approved by the Institutional Animal Care and Use Committee and conducted in accordance with the National Institutes of Health (NIH) Guide for the Care and Use of Laboratory Animals. Animals were euthanized under either isoflurane or chloral hydrate anesthesia according to NIH guidelines.

### Measurement of seizure threshold, susceptibility and severity, and brain water content

Rats that were Nonpreg (n = 9) or Preg (n = 9) were anesthetized initially with isoflurane (1–3% in oxygen) for intubation, electrode placement and instrumentation. Animals were mechanically ventilated to maintain blood gases and pH within normal physiological ranges. Body temperature was monitored with a rectal thermometer and maintained with a heating pad at 37°C throughout the experiment. The dorsal surface of the head was shaved to expose the scalp and silver subdermal corkscrew electrodes (Ambu, Glen Burnie, MD, USA) were implanted under the scalp and secured in place with collodion glue. Electroencephalography (EEG) was recorded unipolarly using a MP150 acquisition system (BIOPAC System Inc., Goleta, CA, USA) to monitor generalized seizure, as previously described [24]. After placement of electrodes, animals were placed in supine position for placement of venous and arterial catheters. Femoral arteries were cannulated to obtain blood samples for blood gas measurements and continuous measurement of arterial blood pressure via a pressure transducer (BIOPAC Systems Inc., Goleta, CA, USA). Femoral veins were cannulated for administration of the anesthetic chloral hydrate and infusion of the chemoconvulsant pentylenetetrazole (PTZ). PTZ was used because it reliably elicits seizure through antagonistic actions at GABA_A_Rs, thereby allowing investigation into the direct involvement of GABA_A_R plasticity in whole brain excitability during pregnancy. After instrumentation, animals were tapered off isoflurane and anesthesia maintained by continuous intravenous infusion of chloral hydrate (41.5 mg / mL in Nonpreg, 50 mg / mL in Preg; 30 μL / min) and seizure threshold measured as previously described [24]. Chloral hydrate was used because it is thought to not depress neural function, and is the preferred anesthetic for studies measuring EEG [32, 33]. Seizure threshold was calculated as the amount of PTZ (mg/kg) required to elicit electrical seizure: T_infusion_ * R_infusion_ * [PTZ] / bw where T_infusion_ is the time of infusion in min, R_infusion_ is the rate of infusion in mL/min, [PTZ] is the concentration of PTZ in mg/mL, and bw is the body weight in kg. Seizure susceptibility scores were also calculated: bw * 10/v, where bw is body weight in grams and v is volume of PTZ infused in μL [21]. Baseline blood pressures were taken 30 seconds prior to PTZ infusion and at seizure onset. EEG was recorded for 30 minutes post-PTZ infusion and seizure severity assessed by counting the number of recurring seizures and calculating the percent of the post-infusion period spent in seizure. Two Nonpreg and three Preg rats were excluded because blood gases were outside of the physiological range. Seizure severity was not assessed in one Preg rat because EEG recordings were not available for the entire 30 minutes due to a technical issue with the ground electrode. After 30 minutes animals were euthanized under chloral hydrate anesthesia by decapitation and brains immediately removed. The cerebral cortex was bisected coronally into anterior and posterior sections just posterior to the M2 vertical branch of the middle cerebral artery. The posterior cerebral cortex was isolated and weighed wet (weight_wet_), then dried in a laboratory oven at 90° C for 24 hours and re-weighed dry (weight_dry_). The posterior brain region was chosen as it is a primary location of edema in women with eclampsia [34]. Percent water content was determined by wet:dry weights using the following formula: (weight_wet—_weight_dry_/weight_wet_) * 100. Brain water content of one Nonpreg rat was not measured because the 24-hour drying time point was unable to be completed.

### Quantification and morphological assessment of microglia

Separate groups of Nonpreg (n = 6) and Preg (n = 8) rats were euthanized under isoflurane anesthesia and brains immediately removed. A 3 mm section (4–7 mm posterior to bregma) was taken of the posterior cerebral cortex and fixed in 10% buffered formalin at 4°C overnight, then transferred to 0.1 M PBS and slices paraffin embedded. Immunohistochemical staining for ionized calcium binding adapter molecule 1 (Iba 1; Wako, Richmond, VA, USA), a marker for microglia was done using standard procedure as described previously [24]. For each brain section, four micrographs of cerebral cortex were captured using an Olympus BX50 microscope at 20X magnification. Each Iba1^+^ cell was assessed by its morphology and activation state ranked using a graded scale from 1 (relatively inactive) to 4 (relatively active) as previously described [24]. To assess microglia, two analyses were performed. First, the percentage of cells in each activation state was calculated for each micrograph and averaged per group. Second, total number of Iba1^+^ cells were counted per mm^2^ and averaged for each group. Two evaluators that were blinded to group performed all morphological assessments.

### GABA_A_R subunit expression by Western blot

Separate groups of Nonpreg and Preg animals (n = 6 / group) were euthanized under isoflurane anesthesia and brains immediately removed. Cerebral cortex and hippocampus were isolated and snap frozen in liquid nitrogen and stored at– 80°C. Cerebral cortex or hippocampus from either Nonpreg or Preg rats were homogenized in homogenization buffer and centrifuged at 100,000 g at 4° C for 30 min. The pellet was resuspended in homogenization buffer containing 1% Triton-X-100 for 1 hour on ice, then centrifuged at 100,000 g at 4°C for 30 min. The supernatant was collected as the membrane fraction as done previously [35]. Protein concentration was determined using a DC Protein Assay (BioRad, Hercules, CA, USA). The supernatant was converted to a gel sample by adding 5X Laemmli sample prep buffer and beta-mercaptoethanol to a final concentration of 280 mM. For determination of membrane protein concentration of GABA_A_R-δ and GABA_A_R-γ2, 35 μg or 25 μg of either cerebral cortex or hippocampus was loaded on a 10% Mini-PROTEAN TGX Precast Gel (BioRad, Hercules, CA, USA) and ran at 30 mA, respectively. The gel was then wet transferred to a polyvinyl difluoride membrane at 90 V for 45 min. The membrane was Ponceau-S stained to ensure that protein was properly transferred. The membrane was blocked with 5% non-fat dried milk in TBS-T for 1.5 hrs at room temperature. The membrane was then rinsed with TBS-T and the primary antibody was applied, anti-GABA_A_R-δ (1:200; Santa Cruz Biotechnology, Dallas, TX, USA) or anti-GABA_A_R-γ2 (1:1000, Novus, Littleton, CO, USA) overnight at 4°C with rocking. The antibodies were removed and the membrane rinsed with TBS-T and secondary antibody was applied, anti-Goat (1:100,000) (Southern Biotech, Birmingham, AL, USA) for GABA_A_R-δ or anti-Rabbit (1:100,000, Promega, Madison, WI, USA) for GABA_A_R-γ2 for 1 hr at room temperature. The secondary antibodies were removed and subsequent TBS-T washes were done. West Pico chemiluminescent substrate (ThermoScientific, Rockford, IL, USA) was applied to the membrane and then exposed to film. β-actin served as a loading control (1:2000, Abcam, Cambridge, MA, USA) and all samples were normalized to a Nonpreg control.

### Drugs and solutions

Chloral hydrate and PTZ were purchased from Sigma Aldrich (St Louis, MO, USA) and made daily in sterile lactated Ringer’s solution. Western blot solutions were prepared as follows: Homogenization buffer contained 50 mM Tris HCl, pH 7.4, 5 mM EDTA, 10 mM EGTA, protease inhibitor cocktail (1:200; Sigma Aldrich, St Louis, MO, USA) and 0.5 mM DTT, and TBS-T contained 15.4 mM Tris-HCl, pH 7.4, 137.0 mM NaCl, 0.1% Tween-20.

### Statistical Analysis

Data are presented as mean ± standard error of mean. All comparisons were made between groups using Student’s t-test and differences were considered significant at p < 0.05.

## Results

### The effect of pregnancy on seizure threshold, susceptibility and severity

**Fig 1:**
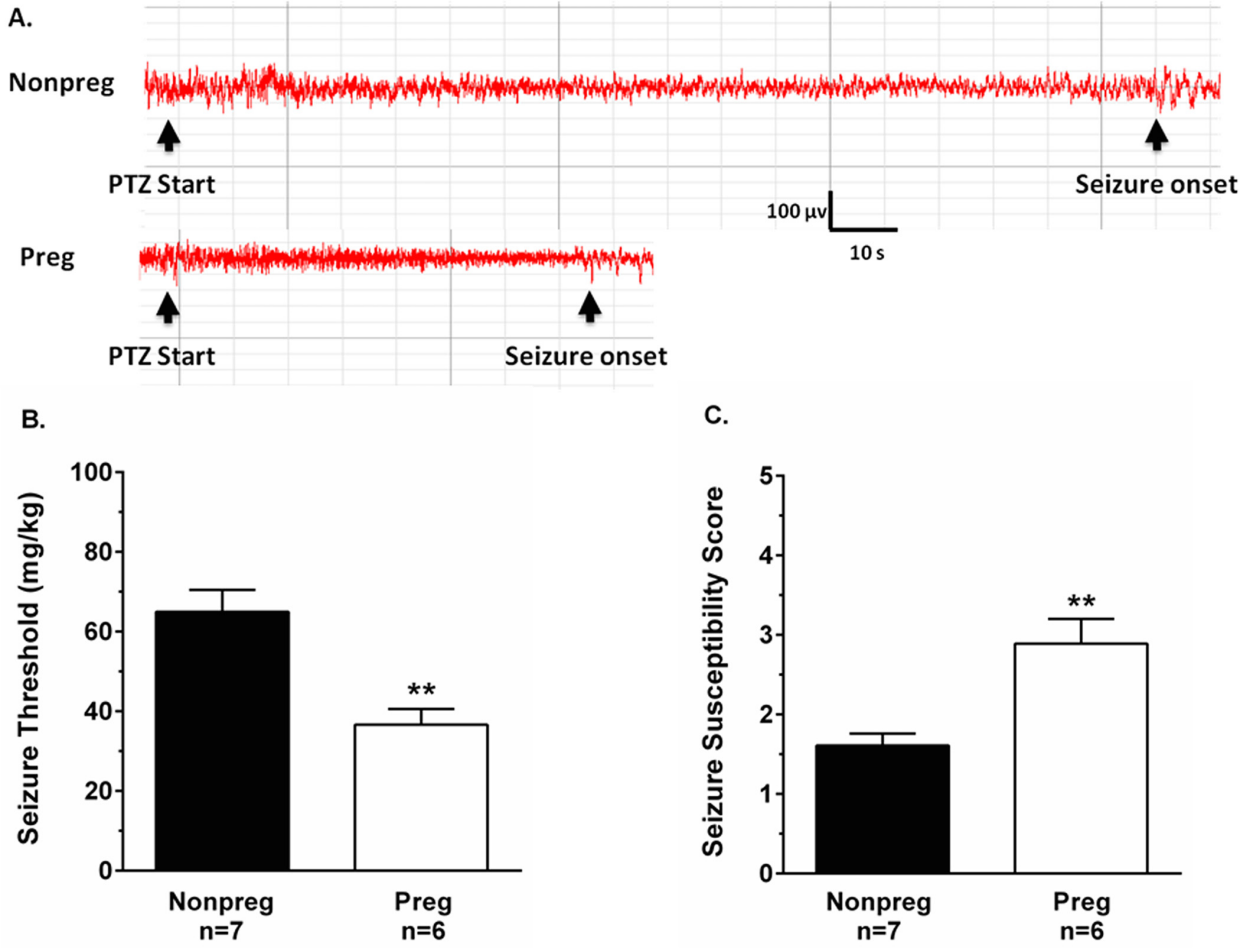
The effect of normal pregnancy on seizure threshold and susceptibility. (A) Representative EEG tracings from nonpregnant (Nonpreg) and late-pregnant (Preg) rats during timed-infusion of pentylenetetrazol (PTZ). Black arrows indicate when PTZ infusion started and the onset of spike-wave discharge indicative of electrical seizure. (B) Seizure threshold was significantly lower in Preg rats compared to Nonpreg rats. (C) Preg rats scored higher on the seizure susceptibility scale compared to Nonpreg rats. ** p < 0.01 vs. Nonpreg by Student’s t-test.

To determine the effect of pregnancy on seizure susceptibility, seizure was induced with PTZ and seizure threshold determined in Nonpreg and Preg rats. Fig 1A shows representative EEG tracings during seizure threshold measurements from Nonpreg (top tracing) and Preg rats (bottom tracing). The black arrowheads indicate when PTZ infusion started and the time of seizure onset. PTZ infusion caused an initial decrease in EEG amplitude in both groups of animals; however, the time to seizure onset was longer in Nonpreg rats compared to Preg rats. When seizure threshold was calculated, the amount of PTZ (mg/kg) required to elicit electrical seizure was significantly lower in Preg compared to Nonpreg rats (Fig 1B). Further, Preg rats had significantly higher seizure susceptibility scores compared to Nonpreg rats (Fig 1C). There were no differences in physiological parameters during seizure threshold measurements between groups, except that, as expected, body weights were significantly higher in Preg compared to Nonpreg rats (Table 1). Baseline arterial blood pressures under chloral hydrate anesthesia were similar between Nonpreg and Preg rats (95 ± 4 vs. 86 ± 4 mm Hg; p > 0.05), and with seizure onset arterial blood pressures remained similar between Nonpreg and Preg rats (85 ± 7 vs. 94 ± 6 mm Hg; p > 0.05).

**Table 1:** Physiological parameters of nonpregnant (Nonpreg) and late-pregnant (Preg) rats under chloral hydrate anesthesia for seizure threshold measurements.

	Nonpreg (n = 7)	Preg (n = 6)
**Body Weight (grams)**	287 ± 7	377 ± 10 **
**Body Temp (** ^**o**^ **C)**	36.3 ± 0.2	36.8 ± 0.2
**Arterial P**_**O2**_ **(mm Hg)**	114 ± 8	111 ± 7
**Arterial P**_**CO2**_ **(mm Hg)**	42.1 ± 1.9	42.8 ± 1.7

** p < 0.01 vs. Nonpreg using Student’s t-test

**Fig 2:**
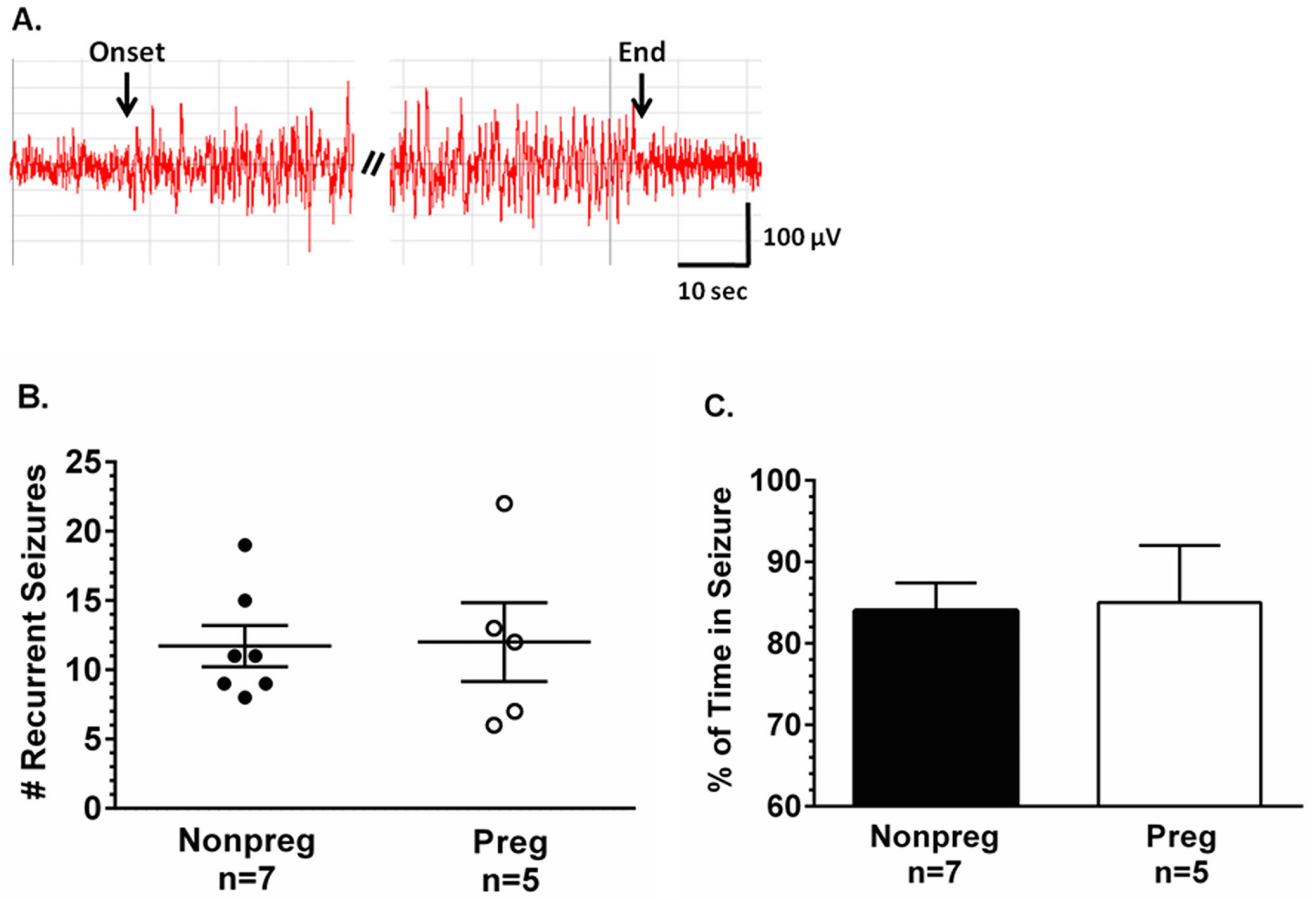
The effect of pregnancy on seizure severity. (A) Representative EEG tracing of a recurrent seizure during the 30-minute post-pentylenetetrazol (PTZ) infusion time period of a nonpregnant (Nonpreg) rat. Left black arrow indicates seizure onset and right black arrow seizure cessation. (B) Number of recurrent seizures in the 30-minute post-PTZ infusion period was similar between Nonpreg and late-pregnant (Preg) rats. (C) The percent of time spent in seizure during the 30-minute post-PTZ time period was similar between Nonpreg and Preg rats.

To determine if there were changes in seizure severity during pregnancy, the total number of recurrent seizures was counted in the 30 minutes post-infusion time period, and the percent of time spent in seizure calculated. Fig 2A shows a representative EEG tracing within the 30 minutes post-PTZ infusion recording time period. Indicated on the tracing is the rapid onset of a recurrent seizure, followed by the rapid cessation of the recurring seizure. There was no change in the number of recurrent seizures in the 30 minutes post-PTZ infusion between groups (Fig 2B). There were also no differences in the percent of time spent in seizure between Nonpreg and Preg rats (Fig 2C).

### The effect of pregnancy on microglial activation and GABA_A_R subunit expression

**Fig 3:**
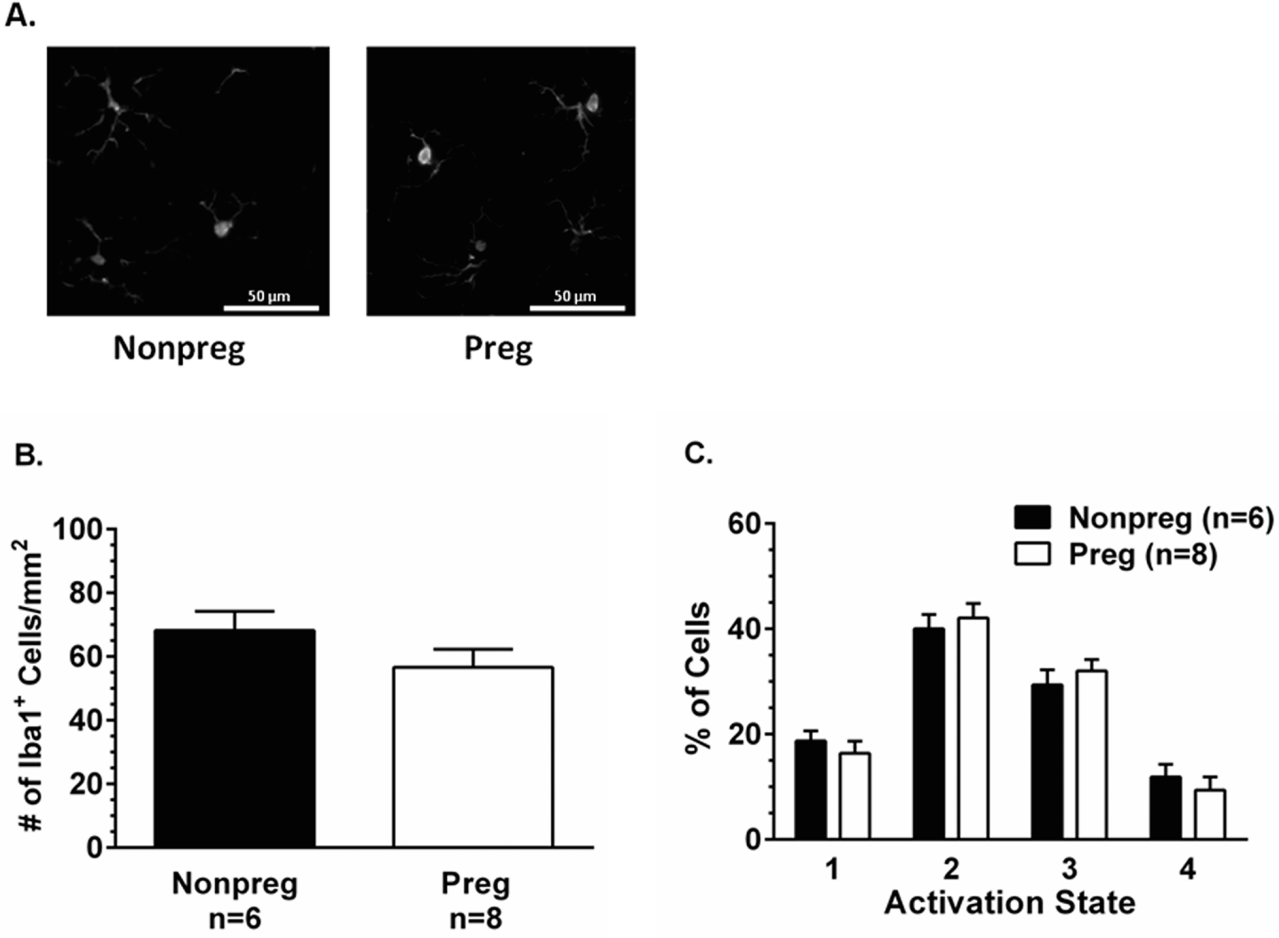
Basal activation state of microglia in cerebral cortex of nonpregnant (Nonpreg) and pregnant (Preg) rats. (A) Representative photomicrographs of Iba 1^+^ microglia in the cerebral cortices of Nonpreg and Preg rats. (B) There was no difference in the number of microglia in the cerebral cortices of Nonpreg and Preg rats. (C) The percent of Iba 1^+^ microglial cells in each activation state was similar in the cortices of Nonpreg and Preg rats.

To determine if normal pregnancy was a state of basal neuroinflammation, we used the microglia-specific marker Iba 1 to morphologically assess the activation state of microglia in the posterior cerebral cortex from Nonpreg and Preg rats. Fig 3A shows representative photomicrographs of Iba 1^+^ microglia in the posterior cerebral cortices of Nonpreg and Preg rats. There was no difference in the total number of microglial cells in the posterior cerebral cortex between groups (Fig 3B). Further, there were no changes in basal microglial activation during pregnancy with no differences in the percent of cells in any activation state between groups (Fig 3C). Thus, it appears that the lower seizure threshold measured in Preg rats was not due to microglial activation.

**Fig 4:**
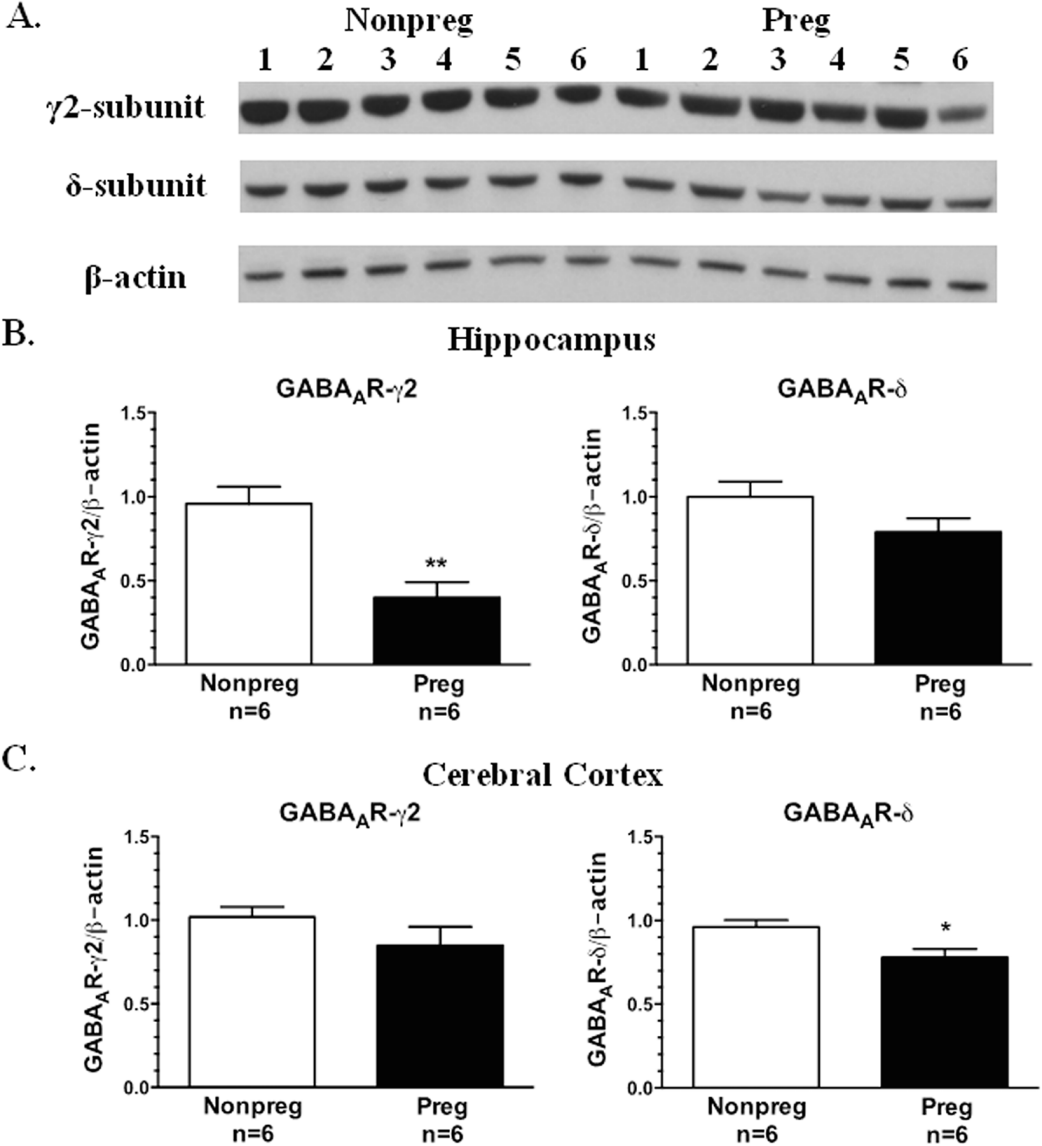
The effect of pregnancy on GABA_A_R δ- and γ2-subunit protein expression in the hippocampus and cerebral cortex. (A) Representative Western blots showing protein expression of the GABA_A_R **γ**2-subunit and δ-subunit in the cerebral cortex of nonpregnant (Nonpreg) and pregnant (Preg) rats. (B) GABA_A_R **γ**2-subunit protein expression was significantly lower in the hippocampus from Preg compared to Nonpreg rats, and GABA_A_R δ-subunit expression trended towards being decreased in the hippocampus of Preg compared to Nonpreg rats. (C) Cortical GABA_A_R **γ**2-subunit expression was similar in Preg and Nonpreg rats, however, GABA_A_R δ-subunit expression was decreased in the cerebral cortex of Preg compared to Nonpreg rats. ** p < 0.01; * p < 0.05 vs. Nonpreg using Student’s t-test.

To determine if pregnancy induced changes in protein expression of GABA_A_R subunits in the hippocampus or cerebral cortex, GABA_A_R δ- and GABA_A_R **γ**2-subunit expression was measured using Western blot, and representative Western blots are shown in Fig 4A. In the hippocampus there was a statistically significant decrease in expression of the GABA_A_R **γ**2-subunit in Preg compared to Nonpreg rats, and a similar trend in GABA_A_R δ-subunit expression (Fig 4B). In the cerebral cortex GABA_A_R **γ**2-subunit expression was similar between groups; however, GABA_A_R δ-subunit expression was significantly decreased in Preg compared to Nonpreg rats (Fig 4C). This pregnancy-induced GABA_A_R subunit plasticity appears to contribute to the decrease in seizure threshold in Preg rats.

### The effect of pregnancy on seizure-induced vasogenic brain edema formation

**Fig 5:**
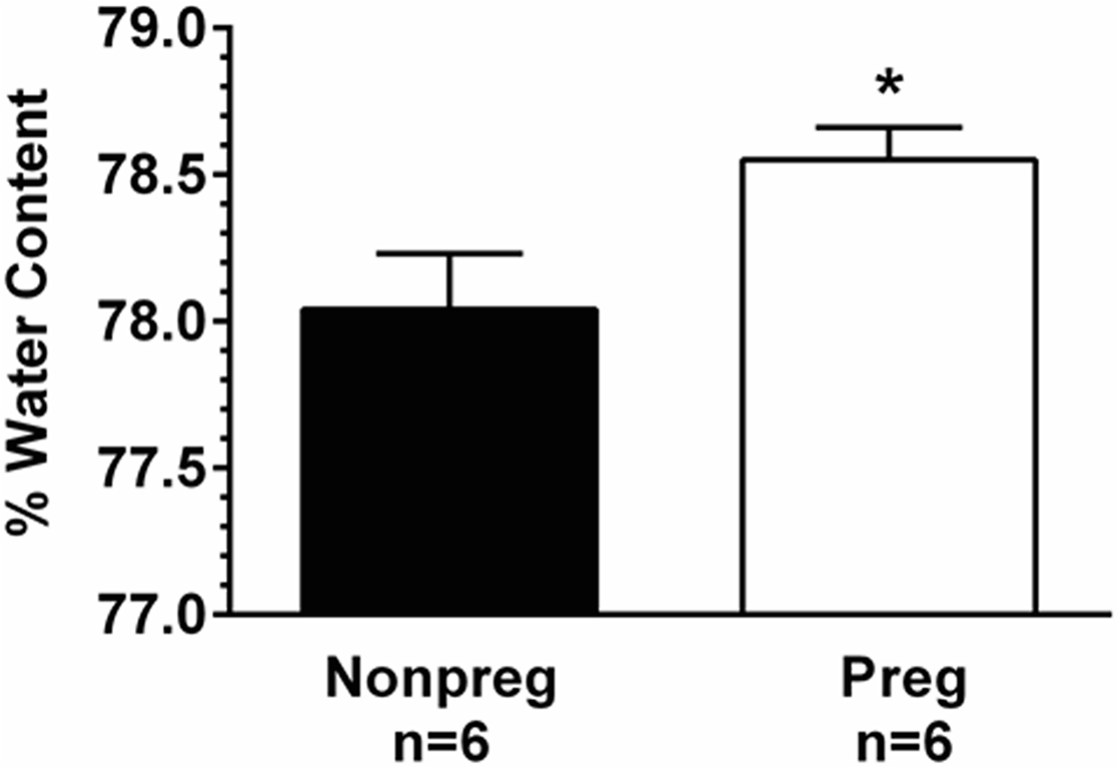
The effect of seizure on vasogenic edema formation in nonpregnant (Nonpreg) and late-pregnant (Preg) rats. Percent water content of the posterior cerebral cortex was significantly higher after seizure in Preg compared to Nonpreg rats. * p < 0.05 vs. Nonpreg using Student’s t-test.

Percent water content of the brain was calculated post-seizure in Nonpreg and Preg rats to determine if there were differences in seizure-induced vasogenic edema formation. The percent of water content was significantly higher in the brains of Preg rats after seizure compared to Nonpreg rats (Fig 5). Thus, the maternal brain seems to be more susceptible to seizure-induced vasogenic edema that the nonpregnant state.

## Discussion

De novo seizure can occur during seemingly uncomplicated pregnancies without preeclamptic symptoms, suggesting that an adaptation to normal pregnancy may increase the sensitivity of the brain to seizure [11, 12]. Therefore, the current study tested the hypothesis that normal pregnancy is a state of increased seizure susceptibility due to basal neuroinflammation and plasticity of GABA_A_Rs. The major finding of this study was that normal pregnancy was a state of increased seizure susceptibility that did not appear to be due to basal neuroinflammation; however, there was decreased GABA_A_R δ-subunit expression in the cerebral cortex and γ2-subunit expression in the hippocampus of pregnant compared to nonpregnant rats that could contribute to the increase in excitability. These findings support the concept that pregnancy may predispose the brain to seizure, whether in seemingly uncomplicated pregnancies or those complicated by preeclampsia, given the maternal brain appears to be in a hyperexcitable state.

The increased seizure susceptibility during pregnancy was not associated with microglial activation. However, we did find decreased inhibitory GABA_A_R subunit expression in the brains of pregnant rats that could affect seizure threshold. GABA_A_R-δ are the main inhibitory neurotransmitter receptors in the brain that are tonically active to provide continuous inhibitory input to neurons and tightly regulate neuronal activity and network excitability [16]. Others have shown that during pregnancy, there is plasticity of GABA_A_Rs in response to elevated endogenous neurosteroids, including the progesterone metabolite allopregnanolone [16, 18, 19, 36]. Allopregnanolone binds to the δ-subunit and enhances the function of GABA_A_Rs [16]. As an apparent compensation to enhanced GABA_A_R function, GABA_A_R subunits downregulate to avoid over-inhibition that maintains a normal state of neuronal excitability [16, 18]. Interestingly, despite high levels of naturally circulating neurosteroids present in pregnant animals in the current study that would be expected to maintain normal excitability, seizure threshold was still lower during pregnancy than the nonpregnant state. Thus, it appears that decreased expression of both GABA_A_R δ- and γ2- subunits during pregnancy were more important for overall excitability than elevated neurosteroids, at least in response to PTZ, a GABA_A_R antagonist.

It is important to note that while we found that seizure threshold was lower in pregnant animals, these animals (and the majority of women) do not have spontaneous seizure during pregnancy. This suggests that there are mechanisms that protect the brain from seizure during pregnancy despite having a hyperexcitable brain, and that de novo seizure during pregnancy may only occur under certain conditions. For example, seizure-provoking factors are circulating late in gestation that cause epileptiform activity when exposed directly to brain parenchyma [37]. However, these factors do not readily gain access to the brain due to the blood-brain barrier (BBB), suggesting the BBB may be a critical interface in preventing seizure during pregnancy. It is possible that the BBB adapts over the course of gestation to protect the maternal brain from seizure-provoking circulating factors. We speculate that failure of the BBB to adapt during pregnancy may be one mechanism by which de novo seizure occurs during pregnancy by allowing passage of seizure-provoking factors into the hyperexcitable maternal brain.

The brain has been shown to be more susceptible to vasogenic edema formation during pregnancy under certain pathologic conditions, such as acute hypertension [28, 29]. The current study shows that the maternal brain was also more sensitive to seizure-induced brain injury, with seizure causing greater vasogenic edema formation than in the nonpregnant state. It is important to note that previous studies have revealed no differences in basal brain water content between pregnant and nonpregnant rats [28]. Thus, the increase in brain water content found in the present study appeared to be due to an effect of seizure. Although edema formation has been found in ~ 90% of women with eclampsia [26, 27], suggesting it to be an underlying cause of the condition, seizure itself can cause edema formation through disruption of the BBB [38–40]. The finding in the current study that the brain is more susceptible to seizure-induced vasogenic edema formation during pregnancy further supports the concept that the brain is more susceptible to injury-induced edema formation during pregnancy. In addition, sensitivity to seizure-induced brain edema during normal pregnancy may explain the presence of vasogenic edema in eclamptic women who experienced de novo seizure without an acute elevation in blood pressure.

There are several potential contributors to increased susceptibility to cerebral vasogenic edema formation during pregnancy. First, if pregnancy were associated with either more severe seizure or seizure-induced acute hypertension, there may be greater brain injury and edema formation. However, there were no differences in seizure severity between pregnant and nonpregnant rats nor did seizure cause a change in blood pressure in either group, making these possibilities unlikely. Second, capillary density increases in the posterior cerebral cortex during pregnancy [41] that may increase the number of sites of BBB disruption during seizure. Lastly, plasma volume increases 50% during pregnancy resulting in a hemodiluted and hyponatremic state that under conditions of BBB disruption such as seizure may drive water into the brain due to decreased osmolality [42]. Regardless of the mechanism, it appears that the maternal brain is more susceptible to vasogenic edema formation during conditions that cause BBB disruption, highlighting the importance of seizure prevention during pregnancy.

In conclusion, understanding pregnancy-related neurophysiological changes may clarify mechanisms by which eclamptic seizure occurs during seemingly uncomplicated pregnancies when there are failures of other protective mechanisms, such as at the BBB or changes in neurosteroid concentrations involved in maintaining steady-state excitability. Further, clarifying the contribution of normal pregnancy to seizure onset could lead to a greater understanding of pregnancy-specific pathologies such as eclampsia. Understanding these conditions may result in development of specific screenings to identify pregnant women who are at risk of de novo seizure, aiding in seizure prevention and specific treatment during pregnancy.
